# A systematic investigation of the relationship between properties of bulk foam and foam in porous media

**DOI:** 10.1038/s41598-023-35278-2

**Published:** 2023-05-17

**Authors:** Abdulrauf R. Adebayo, Suaibu O. Badmus, Sivabalan Sakthivel, Mohamed Gamal Rezk, Rahul S. Babu

**Affiliations:** Center for Integrative Petroleum Research, King Fahd University of Petroleum and Minerals, Dhahran, Saudi Arabia

**Keywords:** Chemical engineering, Chemical engineering, Colloids, Fluids, Characterization and analytical techniques, Hydrology

## Abstract

Bulk foam analysis (static test) is simple and fast, which makes it a cost-effective method for screening and ranking hundreds of surfactants being considered for foam applications. Coreflood tests (dynamic test) can also be used, but it is quite laborious and costly. However, previous reports show that ranking based on static tests sometimes differs from ranking based on dynamic tests. To date, the reason for such a discrepancy is not well understood. Some believe that it may be due to faulty experimental design while some others believe that there is no discrepancy if the right foam performance indices are used to describe and compare the results from both methods. For the first time, this study reports a systematic series of static tests conducted on different foaming solutions (with surfactant concentration ranging from 0.025 to 5 wt%) and duplicated in dynamic tests using the same core sample for all the surfactant solutions. The dynamic test was also repeated on three different rocks of a wide permeability range (26–5000 mD) for each of the surfactant solutions. Unlike previous studies, here multiple dynamic foam indices (limiting capillary pressure, apparent viscosity, trapped foam, and trapped to mobile foam ratio) were measured and compared with the performance indices measured from the static tests (foam texture and foam half-life). Dynamic tests were in total agreement with static tests for all the foam formulations. However, it was observed that the pore size of the base filter disk used in the static foam analyzer can be a potential source of conflicting results when comparing with dynamic test. This is because a threshold pore size exists above which some foam properties (apparent viscosity and trapped foam) significantly decreased compared to the properties before that threshold. Foam limiting capillary pressure is the only foam property that does not show such a trend. It also appears that such threshold occurs above a certain surfactant concentration (0.025 wt%). Apparently, it becomes imperative that the pore size of the filter disk used in the static test and the porous medium used in dynamic tests must be on the same side of the threshold point, otherwise there may be disparity in their results. The threshold surfactant concentration should also be determined. The role of these two factors (pore size and surfactant concentration) requires further investigation.

## Introduction

Foam has many industrial applications including food, pharmaceuticals, the oil and gas industry, etc. In petroleum engineering applications, aqueous foam is mainly used to reduce gas mobility during gas injection projects, and hence improve oil recovery. Furthermore, foam is used in CO_2_ geo-sequestration applications due to its high capabilities to reduce gas relative permeability and gas trapping^1^. Aqueous foam is formed by dispersing gas into a continuous surfactant solution. The gas–liquid interface is stabilized during foam generation due to surfactant aggregation at the interface. At the pore scale, the generated foam consists of flowing gas and trapped gas. The former occupies large pores which have a low resistance to flow, while the latter occupies small to medium pore spaces^2^. The foam bubbles are separated by a continuous liquid film called lamellae, which causes an increase in the viscous force during flow by creating a high resistance to the gas flow^3^.

The generated foam must be strong and stable to achieve the intended results. Different types of surfactant and nanoparticles can give different degrees of foam strength and stability. For this reason, many commercial surfactants and/or nanoparticles solutions must be screened and ranked for their suitability for application in specific porous media and environmental conditions. Such large-scale screening can be done on bulk foam (static foam analysis) or micro foam flowing in porous media (coreflood or dynamic foam analysis). Coreflood is laborious and costly. On the other hand, bulk foam analysis is simple and fast, which makes it a cost-effective method for screening tens or hundreds of surfactants being considered for foam applications. However, there have been some reports on the lack of relationship between static foam and dynamic foam analysis^4^. The relationship between static foam and dynamic foam can be of two types. The first is comparing the ranking of foam formulations (based on some defined indices) from static tests with the ranking from dynamic tests. The second is a direct comparison of foam performance (strength and stability) of a given foam formulation as measured from a static test compared to measurement in dynamic tests. For both cases, there should be a similarity in the results if the methodology used is correct and the right indices are compared. For the case of ranking surfactant formulations, Mannhardt et al.^5^ used different experimental methods (coreflood, bulk foam, micromodel observations, and interfacial parameters) to evaluate and rank the performance of six different foam formulations. They found out that each experimental method gave a different performance ranking for the six foam formulations tested, making it difficult to screen or choose the appropriate foam formulation. Similar disparity was also reported by Jones et al.^6^. These researchers then concluded that the evaluation of foams for field applications must be done using Core flood experiments at reservoir conditions. For direct comparison, some researchers reported that there was no correlation between the foam stability test conducted in static test and those conducted in dynamic tests^7,8^. On the other hand, some other researchers reported a good correlation between the two^9–14^. The cause of such disparity is not clear. Some authors simply attribute it to the fact that foam behavior in porous media is different from foam behavior in its bulk form. Certainly, foam flow in porous media experiences many influencing factors that affect foam behavior in porous media. These include surfactant concentration, injection rate, gas fraction (foam quality), temperature, salinity, the presence of impurities (e.g., crude oil), mechanical shear and stretching as they pass through pores and throats, adsorption on rock surfaces, and then of course the properties of the porous media. Different surfactants will respond differently because of these factors. The desired surfactant must have the least adsorption tendency on rock surface and must also be able to generate strong and stable foam. Hence, adsorption, strength, and stability are the main criteria for screening and selecting surfactant for any foam project. Bulk foam analysis can explicitly give an indication of foam stability through ‘half-life’ measurement while foam strength can be evaluated based on the bubble texture observed through microscopic images of the bulk foam. Adsorption tests can then be conducted on rock samples from the reservoir rock of interest. For fairness in ranking among many surfactants, bulk foam analysis is done on all the surfactants at the same fluid and experimental conditions (temperature, brine salinity, surfactant concentration). The Coreflood test can also give an indication of foam strength and stability which are inferred from its limiting capillary pressure, apparent viscosity, and resistant factor. Similarly, for fairness in ranking, coreflood tests must be conducted in the same rock type for all the surfactants and at the same experimental and fluid conditions (brine salinity, surfactant concentration, temperature, pressure, foam quality, injection rate, etc.). If after this, disparity occurs in the ranking, then attention may be drawn to experimental errors or in technical definition of what the performance indices used in both methods represent. There is therefore the need to carry out a well-designed and systematic study to investigate whether results from static foam tests and dynamic tests correlate. To the best of the authors’ knowledge, this study is reporting for the first time, a one-to-one comparison between multiple static tests (from a single static foam analyzer) and multiple dynamic tests (from the same core sample) for a wide range of foam formulations/strength and a wide range of core properties. Many performance indices were also obtained from the dynamic tests and compared with bulk test performance indices namely half-life, foam volume, and bubble texture. Also, rather than investigating different types of surfactants, here the same surfactant is used but with varying concentration, since different surfactant concentration generate foam of different strength, texture, and stability^15^.

## Methodology

The methodology section is divided into bulk foam analysis, rock characterization, and core flooding.

### Bulk foam analysis

A conventional static foam analyzer was used to measure foam height and foam half-life as a function of time at room conditions (23 °C). The static foam analyzer consists of a vertical transparent glass column (25 cm high and 4 cm inner diameter) and a filter disc of pore size ranging from 40 to 100 µm at the base. A volume of 50 cc of the bulk fluid (various concentrations of surfactant solutions in seawater) was placed in the glass column. The bulk foam was then generated by sparging nitrogen gas through the filter disk mounted below the bulk fluid at a gas rate of 0.3 L per minute for the duration of 12 s. Foam volume/height, half-life/stability, and microscopic images/texture were then measured over time. The surface tension of the surfactant solutions was also measured using a dynamic contact angle tensiometer. The details of the measurement apparatus and procedure are provided in a previous study^16^.

### Rock and fluid characterization

Three outcrop rock samples covering both sandstone and carbonate with a wide permeability range were acquired and cut into cylindrical plugs whose dimensions and petrophysical properties are listed in Table 1. The pore structure of the rock samples was analyzed using a high-resolution micro-CT scanner on the end pieces extracted from the edge of the rock samples. The details of the procedure and experimental apparatus are provided in a previous paper^17,18^. A pore network modeling conducted on the micro-CT images generated information about the average pore size and average pore throat size of the rock samples.

**Table 1:** Summary of rock samples.

Rock properties	Rock samples		
	AC	B	IDG
Helium porosity (%)	26.8	22.1	29.2
Absolute helium perm. (mD)	26	278	5000
Length (cm)	10.44	9.35	10.41
Diameter (cm)	3.78	3.78	3.77
Rock type	Chalk	Sandstone	Sandstone
Calcite (%)	98	–	–
Quartz (%)	–	92	80
Kaolinite (%)	–	7	–
Montmorillonite (%)	2	1	11
Muscovite/Illite (%)	–	Trace	1
Feldspar (%)	–	–	8

Nuclear magnetic resonance relaxation measurements were also conducted on the samples when they were 100% saturated with brine, using a low field (2 MHz) benchtop NMR apparatus. Such measurements provide information on the pore size distribution of the rocks in terms of the relaxation rate of the hydrogen nuclei of the fluid in the different pores.

Porous plate measurements were conducted on the rock samples to obtain the capillary pressure distribution for the different rock samples. Such capillary pressure curves are needed to identify the limiting capillary pressure at which foam begins to coalesce or rupture.

A synthetic brine with a salt concentration of 67,479 ppm was prepared in the laboratory (Table 2). Different concentrations of a nonionic surfactant (Triton X-100) were then dissolved in the brine to form the surfactant solutions. A non-ionic surfactant was used because our previous study showed that it outperformed other types of surfactants^11^. Nitrogen gas with 99.9% purity was used as the gas phase in all experiments. Brine with different surfactant concentrations was prepared in batches namely: 0.025 wt%.; 0.05 wt%.; 0.1 wt%.; 0.5 wt%.; 1 wt/vol %.; 2.5 wt%.; and 5 wt%.

**Table 2:** Brine composition.

Salt	Weight (g/L)
NaHCO_3_	0.17
Na_2_S0_4_	6.34
NaCl	41.17
CaCl·2H_2_0	2.39
MgCl_2_·6H_2_0	17.42
Total	67.48

### Coreflood procedure

For a given batch of surfactant solution, the rock samples were initially saturated with the solution by a vacuum saturation method. Coreflood experiments were conducted at an atmospheric temperature of 23 °C, confining pressure of 2200 psi, and back pressure of 1450 psi. Before the gas injection, the rock sample was further injected with about 2 pore volumes of the surfactant solution at an injection rate of 0.5 cm^3^/min to ensure full saturation with surfactant and to reduce the impact of surfactant losses by adsorption to the rock surface. Differential pressure transducers were installed on the Coreflood apparatus to measure and record pressure data on the computer station at an interval of 5 s. Electrical resistivity ports were also installed on the core holder to monitor the electrical resistivity of the rock sample and recorded on the computer. The adopted coreflood procedure for foam injection was described in our previous articles and allowed for trapped and mobile foam saturation to be monitored continuously^17,19,20^. In this method, about 0.1 pv of gas was injected into the rock sample initially saturated with surfactant solution at a rate of 0.5 cm^3^/min and followed by a continuous brine (containing surfactant) injection until a steady state was reached. The trapped gas at this steady state is then measured through resistivity measurement. This is then followed by a second cycle, where a higher pore volume of gas (e.g., 0.2 pv) is injected and followed by a continuous brine injection until another steady state and the new trapped gas saturation and mobile foam were measured. The experiment was stopped when there were no significant changes in resistance and pressure drop measurement with an increase in the PV of gas injected into the rock sample. Here, the global steady of trapped gas was attained.

## Results and discussions

### Bulk foam

**Figure 1:**
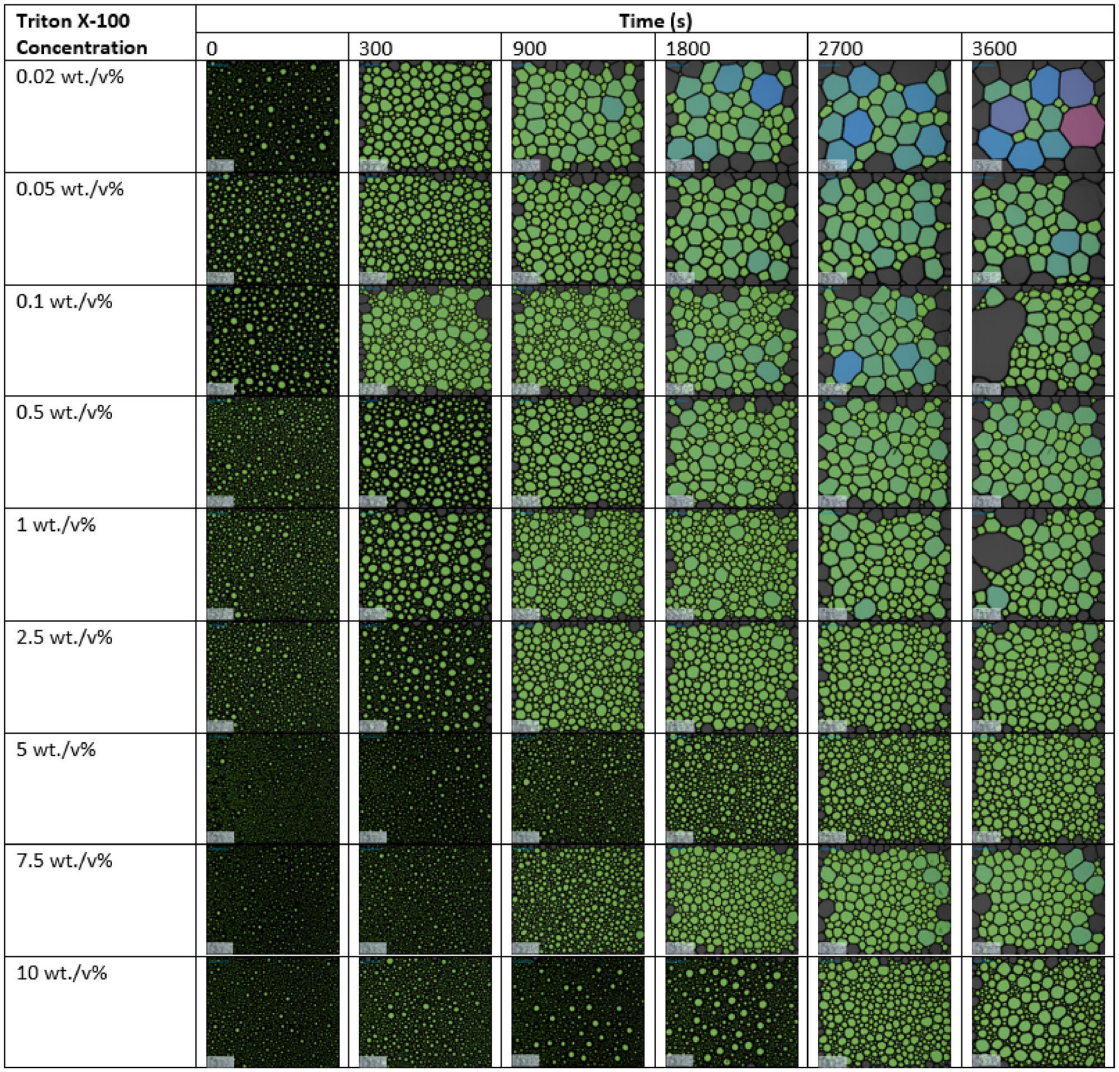
The texture of bulk foam at different time steps after bulk foam was generated (0–3600 s). The texture is shown for different surfactant concentrations ranging from 0.02 to 10 wt%.

Figure 1 shows the foam texture analysis at different concentration of surfactant solutions and at the different elapsed times after they were generated in the static foam analyzer. Moving from left to right for each surfactant concentration in Fig. 1, foam bubbles collapse with elapsed time due to Ostwald ripening or bubble coalescence. This results in larger bubble sizes and smaller bubble density (numbers) as the foam ages and its height decreases. However, an increase in surfactant concentration (moving from top to bottom in Fig. 1) at each elapsed time, results in a better foam texture. The effect of surfactant concentration appears to be optimum at a concentration of 2.5 wt%. Hence, there was no substantial improvement in foam texture beyond this concentration.

**Figure 2:**
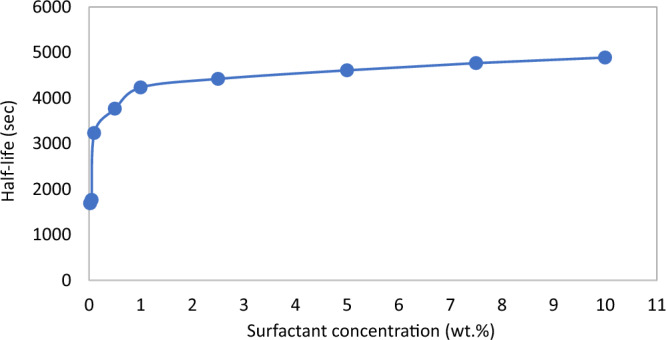
Foam half-life versus surfactant concentrations.

**Figure 3:**
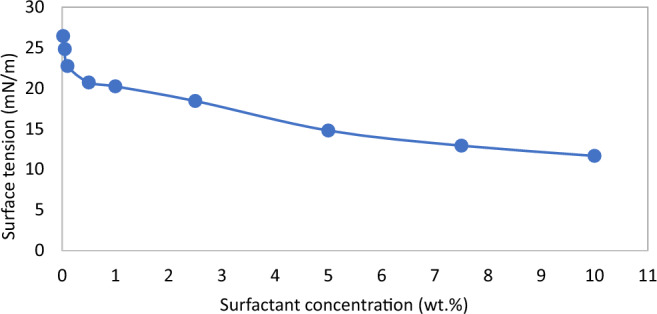
Surface tension of surfactant solution at various concentrations.

In terms of the foam stability, as shown in Fig. 2, foam half-life increases with an increase in surfactant concentrations. However, at concentration above 2.5 wt%, the increase of the half-life is not so significant. Similarly, in Fig. 3, the measurement of the surface tension was very steep up to 2.5–5 wt%, after which the reduction was founds to be gradual. It is clear from Figs. 2 and 3 that an asymptote has not been reached, which indicates that slight changes in foam properties occur as surfactant concentration is increased. Nonetheless, an optimum surfactant concentration value is required, which is the concentration beyond which the properties of the foam do not change significantly to justify the associated cost of increasing the concentration. From the analysis of the bulk foam in this study, 2.5 wt% appears to be the optimum concentration. The findings from the investigation of optimum surfactant concentration for foam performance in porous media are presented in the next section.

### Foam in rock samples

**Figure 4:**
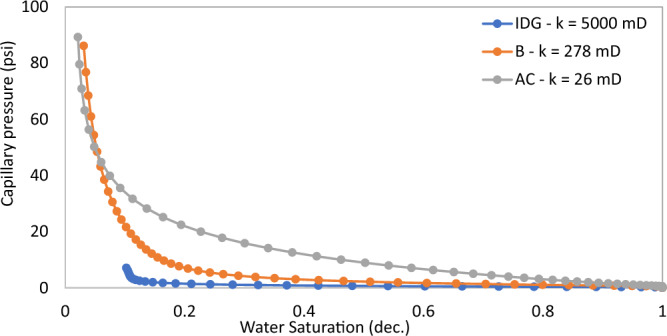
Capillary pressure curves.

**Figure 5:**
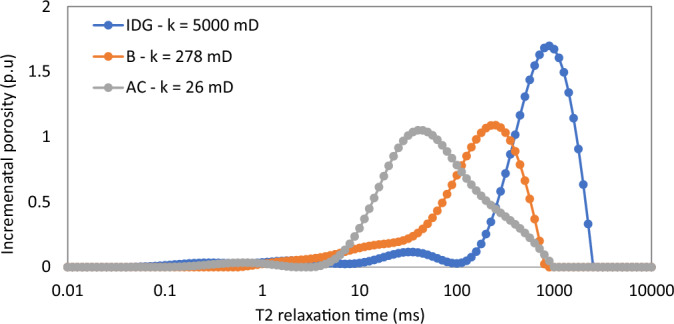
NMR T_2_ relaxation curves.

First, the pore characteristics of the rock samples are presented in terms of their capillary pressure curves (Fig. 4) and NMR T_2_ relaxation distribution (Fig. 5). Table 2 also summarizes other data on pore structure based on micro-CT image-based pore network models and XRD analysis. It is obvious from these figures that the three rock samples are significantly distinct from one another in terms of porosity, permeability, capillary pressures, and pore size distribution. This paves the way for a more holistic study of the impact of a wide range of rock pore structures on foam properties. Sample IDG has a low capillary pressure regime because of the large pore sizes (as illustrated by the long T_2_ relaxation in Fig. 5), while sample AC has the highest capillary pressure because it has the lowest pore size (short T_2_ relaxation). Sample B lies between the two extremes in all definitions of pore characters as shown in Table 3.

**Table 3:** Summary of rock properties and pore geometry.

Pore character	Rock samples		
	AC	B	IDG
Helium porosity (%)	26.8	22.1	29.2
Absolute helium perm. (mD)	26	278	5000
Average pore radius (µm)	4.06	5.67	8.87
Average throat radius (µm)	2.72	3.5	5.89
Average aspect ratio	1.5	1.62	1.5
T_2LM_ (ms)	60	117	571

Trapped and mobile foam saturation of the rock samples were estimated from measured resistivity values by using Archie’s equation (Eq. 1).

1 $$ S_{w} = \left( {\frac{{R_{0} }}{{R_{t} }}} \right)^{1/n} $$

where ${S}_{w}$ is the in-situ water saturation in the rock sample (in fraction), ${R}_{0}$ (ohm-m) is the electrical resistivity of the rock when it is 100% saturated with brine (with dissolved surfactant), ${R}_{t}$ (ohm-m) is the resistivity of the rock at partial water saturation, and ‘n’ is the saturation exponent, a parameter derived during a porous plate resistivity index experiment. Since only two phases were flowing through the porous medium, i.e., liquid and gas, gas, or foamed gas saturation (${S}_{g}$) at any instance is estimated with Eq. (2).

2 $${S}_{g}=1- {S}_{w}$$

**Figure 6:**
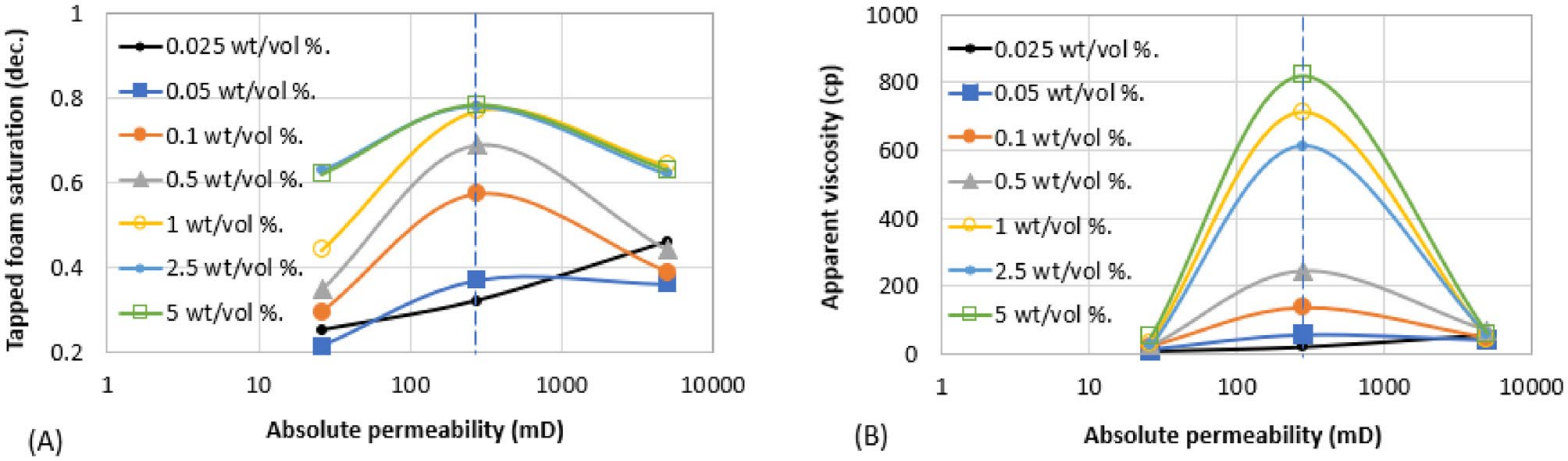
Effect of surfactant concentration on (**A**) trapped foam (**B**) Apparent viscosity of foam, for different permeability.

**Figure 7:**
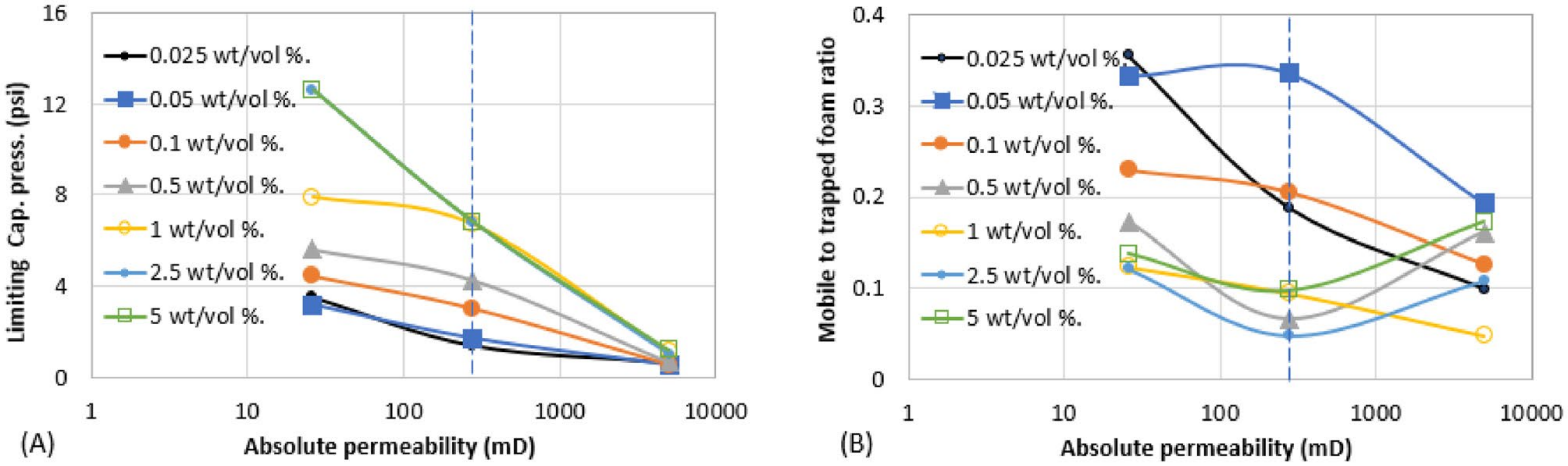
Effect of surfactant concentration on (**A**) limiting capillary pressure of foam (**B**) mobile-to-trapped foam ratio, for different permeability.

**Figure 8:**
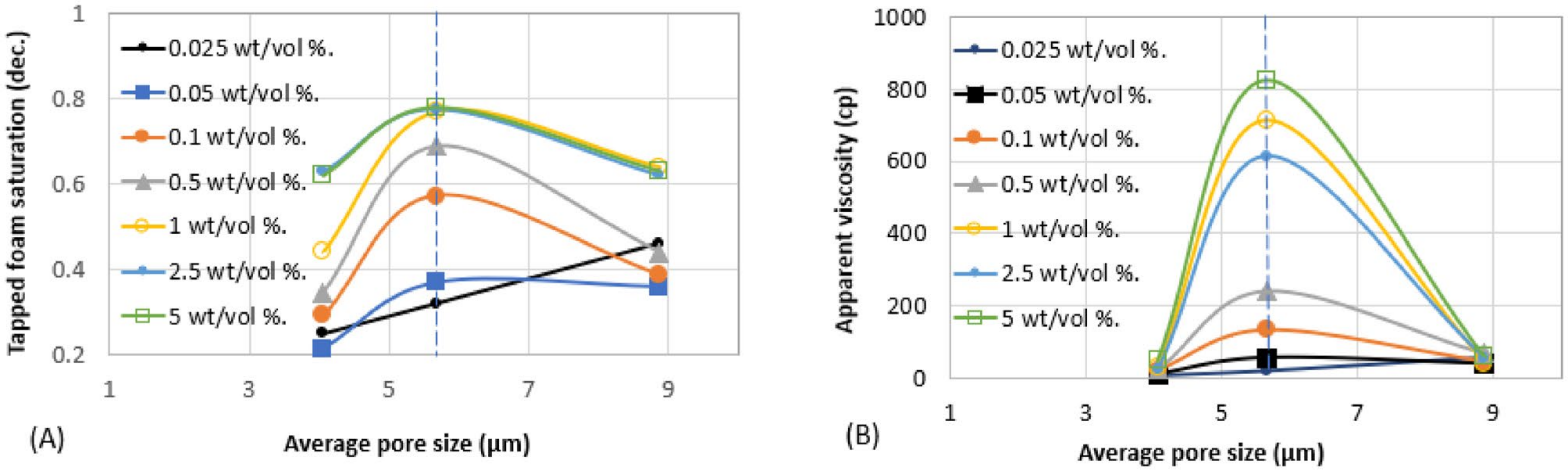
Effect of surfactant concentration on (**A**) trapped foam (**B**) mobile-to-trapped foam ratio for different pore sizes.

**Figure 9:**
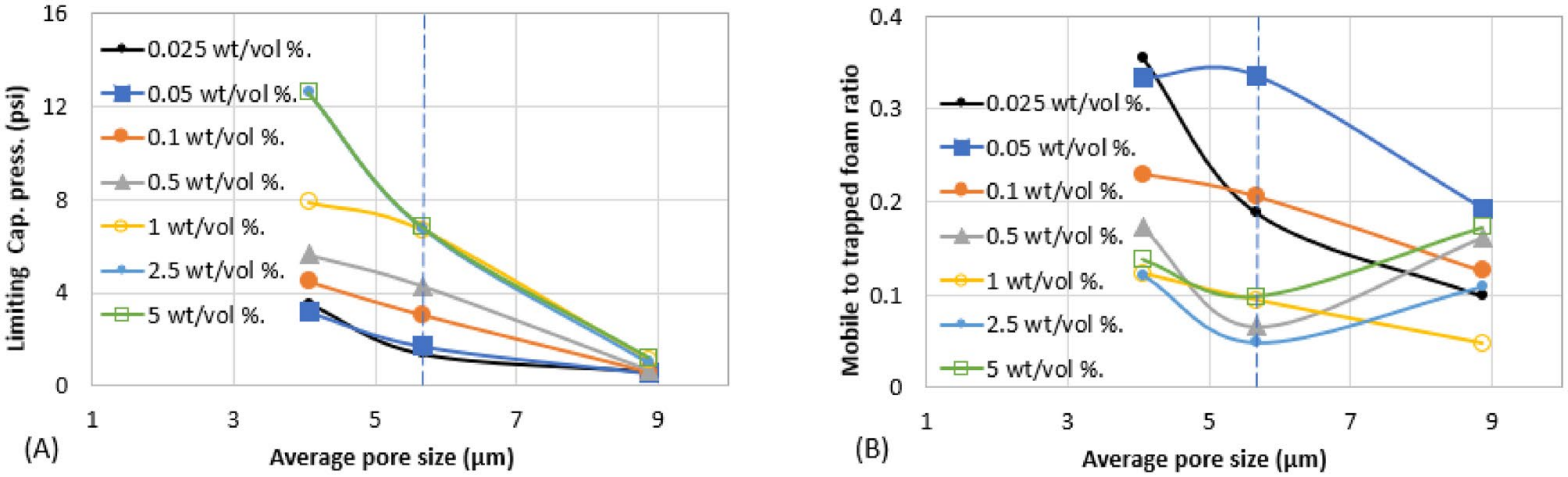
Effect of surfactant concentration on (**A**) limiting capillary pressure of foam (**B**) mobile-to-trapped foam ratio, for different pore sizes.

**Figure 10:**
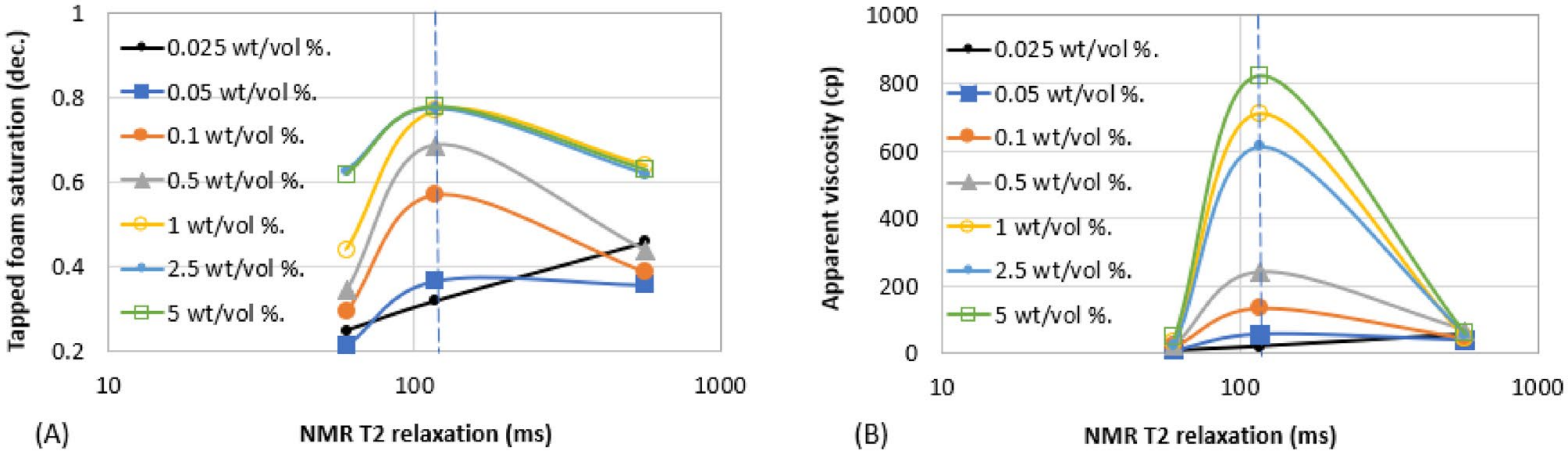
Effect of surfactant concentration on (**A**) trapped foam (**B**) apparent viscosity, for different T_2LM_.

**Figure 11:**
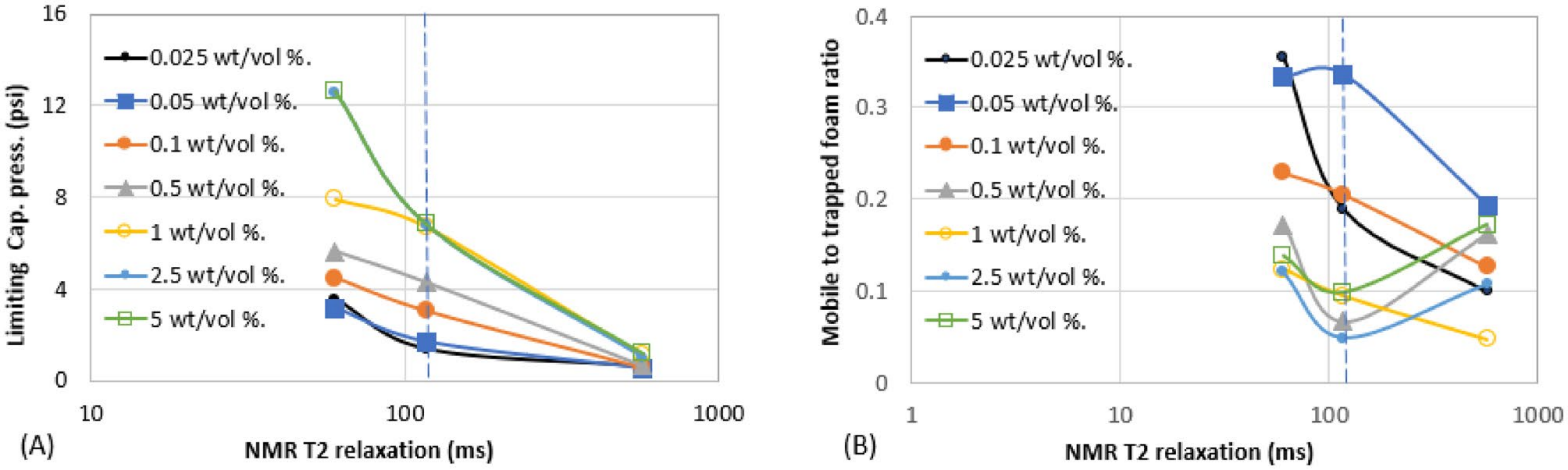
Effect of surfactant concentration on (**A**) limiting capillary pressure of foam (**B**) mobile-to-trapped foam ratio, for different T_2LM_.

Figures 6, 7, 8, 9, 10 and 11 show different foam properties (trapped foam, apparent viscosity, limiting capillary pressure, mobile-to-trapped foam ratio) versus surfactant concentration in all three rock samples. These foam properties changed as surfactant concentration increased to different degrees.

In terms of the effect of surfactant concentration on given foam properties, a good agreement was observed among all measured bulk foam properties and foam properties in the rock samples. All static and dynamic foam properties were improved with an increase in surfactant concentration (foam strength). However, disparity occurs when the effect of pore properties was considered in the comparison, which suggests that the comparison of foam properties in different rocks must be done cautiously.

Figures 6, 7, 8, 9, 10 and 11 show how surfactant concentration affects trapped foam, apparent viscosity, and limiting capillary pressure in a variety of rocks with a wide range of pore character: permeability range of 26–5000 mD; average pore size of 4–9 µm; average throat size of 2.7–6 µm. The saturation of trapped foam and consequently the apparent viscosity of foam increase for every increase in surfactant concentration (by extension for every increase in foam strength) and with each rock pore character, represented by a data point in the figures. However, the character of the pores above a threshold value has a reverse effect on the foam properties as seen in Fig. 6. Based on these figures, it appears that an optimum permeability value exists somewhere between 278 and 5000 mD at which foam properties reverse its trend from the initial trend. Interestingly, the reversal (or drop in) trapped foam saturation and apparent viscosity only occur when the surfactant concentration is higher than 0.025 wt%. As the surfactant concentration increases, the severity of the reversal increases (i.e., the curve becomes more concave). The exact reason for this requires a more comprehensive study. There is almost a complete overlap between surfactant concentration of 2.5 wt and 5 wt%, for foam trapping (Fig. 6) and limiting capillary pressure (Fig. 7).

The similarity between the behavior of foam trapping and the apparent viscosity of foam shows that trapped foam saturation is mainly governed by the apparent viscosity of foam.

The limiting capillary pressures of foam also increase when surfactant concentration increases (Fig. 7A). However, with respect to changes in permeability, the limiting capillary pressure of foam differs from other foam properties (i.e. trapped foam saturation and its apparent viscosity). The limiting capillary pressure decreases as permeability increases. This is expected because the capillary pressure regimes in high-permeability rocks are not as high as in lower-permeability rocks. Hence, since foam limiting capillary pressure is the most direct measure of foam strength^12^, foam is stronger in low-permeability rock than in high-permeability rock, and with an increase in surfactant concentration, it further increases the strength of generated foam (Fig. 7A). There is also no reversal in the limiting capillary pressure trend as was observed in the case of trapped foam saturation and apparent viscosity in Fig. 6.

Since increasing surfactant concentration increased trapped foam saturation (Fig. 6A), it is then expected that mobile foam saturation decreases as surfactant concentration increases as shown in Fig. 7B. Similarly, for the same reason, the mobile-to-trapped foam decreases with an increase in permeability, with a similar reversal at the same threshold permeability value in Fig. 6A.

Based on the results in Figs. 6 and 7, it can be suggested that trapped foam saturation directly affects apparent viscosity and mobile-to-trapped foam ratio. Hence, trapped foam saturation or any of its dependent variables can be used to compare foam performance in rocks of the same pore character. However, it cannot be said that foam strength increases with increased trapping when comparing two different rock pore geometry since trapping and limiting capillary pressure have a different correlation with the change in pore geometry.

It was earlier shown that foam with large bubbles corresponds to weak foam based on bulk foam analysis in Fig. 1. Also, rocks with large pores, large throats, and high permeability enhance the formation of large bubbles (large foam texture) since foam assumes the structure of the pores where they are generated. The tendency of weak (coarse) foam to flow is also lower because of low differential pressure and low flow velocity across high permeability rock^19,21,22^. Hence, they remain trapped due to insufficient pressure drop present in high permeability rocks. As a result, trapped foam saturation is expected to rise with increased permeability and vice versa. The limiting capillary pressure of foam also decreases with an increase in permeability (Fig. 7), a confirmation that high permeability rocks generate weaker foam than lower permeability rocks. It therefore becomes difficult to judge if higher trapped foam saturation in one rock sample relative to another is an indication of stronger foam or weaker foam.

The aforementioned foam properties were plotted with other rock pore characters (average pore size, average throat size, and the log mean of T_2_ relaxation). These foam properties have similar responses to the variation in these pore characters as shown in Fig. 8 (Trapped foam and apparent viscosity versus pore size); Fig. 9 (limiting capillary pressure and mobile-to-trapped foam ratio versus pore size); Fig. 10 (trapped foam and apparent viscosity versus NMR T_2_); and Fig. 11 (limiting capillary pressure and mobile-to-trapped foam ratio versus NMR T_2_). Average pore size can also be represented in terms of the logarithmic mean of NMR T_2_ relaxation since T_2_ relaxation has a one-to-one relationship with pore size. The larger the pore size, the longer the T_2_ relaxation time.

**Figure 12:**
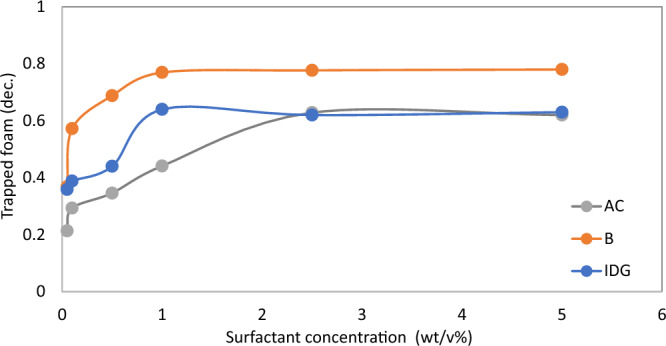
Trapped foam saturation versus surfactant concentration for three samples.

**Figure 13:**
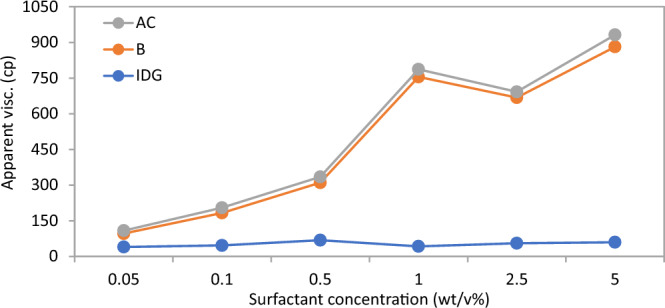
Apparent viscosity of foam versus surfactant concentration for three rock samples.

**Figure 14:**
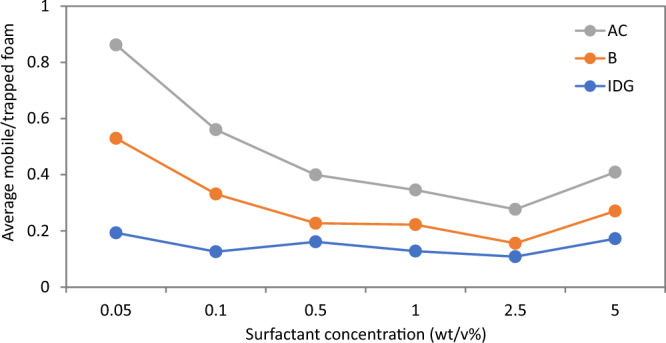
Average mobile-to-trapped foam ratio versus surfactant concentration in three rock samples.

**Figure 15:**
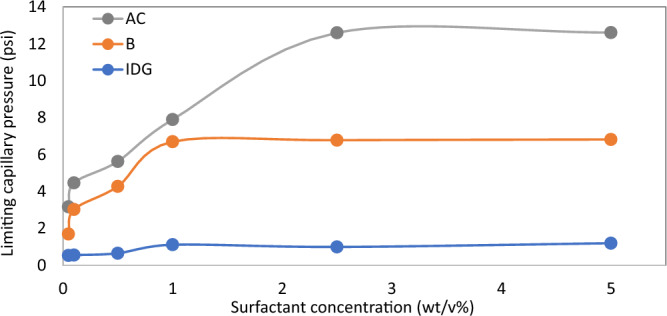
Limiting capillary pressure of foam versus surfactant concentration in three rock samples.

To determine the optimum surfactant concentration for foam in porous media, each of the foam properties was plotted against surfactant concentration for the three rock samples tested (Figs. 12, 13, 14 and 15). From the results, it can be concluded that the optimum surfactant concentration for foam trapping, apparent viscosity, mobile-to-trapped foam ratio, and limiting capillary pressure appears to be 1 wt%, in sample B and IDG, while that for sample AC appears to be 2.5 wt%. This optimum value is similar for bulk foam, which is 2.5 wt%.

### Comparison between bulk foam and foam in porous media

The findings above have a significant implication when comparing foam properties measured by a different group of researchers or measured using different experimental procedures. Static foam analyzers have a porous disk at their base with varying permeability or pore size distributions. For example, the pore size distribution of the porous disk in the static foam analyzer used in this study is 40–100 µm. If another group of researchers working elsewhere with a foam analyzer (with an average pore size of say 4 µm) conduct a similar experiment with the same surfactant type and concentration, they may not be able to replicate the results. This is because both research groups use static foam analyzers with different base porous disks, whose individual pore sizes fall on two different extremes of the threshold pore size/permeability values reported in Figs. 8 and 10. There is a variety of specifications of static foam analyzers available, with the pore sizes of the base porous disk (filter) ranging from 0.5 to 100 µm^11,12,16,23^, which makes the experimental conditions differ, especially the capillary pressure. Some other foam analyzers do not use a porous disk to generate foam, but by mere mixing gas and surfactant solution at defined speeds like in a blender^9,24^. Since the foam texture or bubble size is dependent on the pore size of the porous discs^25^ or the mixing speed, the rate of foam decay, half-life, and other foam properties can defer among the foam analyzers for the same foam formulation. It is therefore a good practice for researchers to report more information about the static foam analyzer used in the reported tests such as the average pore size of base porous disk, the procedure for generating foam in the foam column (gas sparging or blender method), gas bubbling rate through the disk (for gas sparging foam analyzer), the stirring rate (for a blender type of foam analyzer). There is also a concern about whether static foam tests measure foam stability only or whether it gives an indication of foam strength^26^. Hence, for a fair comparison between static foam and dynamic foam, the porous disk or filter in the static foam analyzer must have a similar pore geometry as the rock samples used in core flooding experiments.

## Conclusions

In this study, we investigated and compared the properties of both bulk foam and foam in porous media for different foam solutions. To do so, static foam analysis was conducted using a conventional static foam analyzer. Foam volume/height, half-life/stability, and microscopic texture were measured over time. Core flooding experiments were also conducted to obtain various foam properties in porous media. Based on the results of this study and under the experimental conditions, the following main conclusions are drawn:

- Bulk foam performance is in total agreement with foam performance in porous media when the appropriate performance indices are compared.
- Increasing surfactant concentration increased bulk foam half-life and foam texture until an optimum surfactant concentration above which increasing the concentration did not yield a significant change in foam properties. Similarly, increasing surfactant concentration increased trapping, apparent viscosity, and limiting capillary pressure of foam in porous media until an optimum surfactant concentration. Further increase in concentration did not show a significant change in foam properties. The optimum surfactant concentration in the static test was 2.5 wt%. while that of dynamic test was 2.5 wt% for one of the core samples and 1 wt%. for the other two samples.
- At a surfactant concentration above 0.025 wt%, trapped foam and apparent viscosity of foam increased in porous media with larger pore size/permeability until above a certain threshold value where foam trapping and apparent viscosity decreased. The reverse trend did not occur at a surfactant concentration of 0.025 wt% and for the foam limiting capillary pressure. The reason for this is not clear and requires further studies.
- Where comparison is to be made between static test and dynamic test, adequate information must be provided about specifications of the static foam analyzer used and the procedure for generating foam in it. To have a match between the two methods, the pore size of static foam analyzer must match that of coreflood sample. The flow rate of gas used in both experiments must also match. Researchers must therefore report more information about each method such as the average pore size of base porous disk, the procedure for generating foam in the foam column (gas sparging or blender method), gas bubbling rate through the disk (for gas sparging foam analyzer), the stirring rate (for a blender type of foam analyzer). It is also recommended that coreflood tests for screening purposes be conducted on the same rock type.

## Data Availability

The datasets generated during and analyzed during the current study are available from the corresponding author on reasonable request**.**
